# Comparative study of Mg/Al-LDH and Mg/Fe-LDH on adsorption and loss control of 2,4-dichlorophenoxyacetic acid

**DOI:** 10.1007/s44307-024-00055-3

**Published:** 2025-01-21

**Authors:** Zeyuan Zhang, Liangjie Tang, Jing Luo, Jinfang Tan, Xiaoqian Jiang

**Affiliations:** School of Agriculture and Biotechnology, Sun Yat-Sen University, Shenzhen, Guangdong 518107 People’s Republic of China

**Keywords:** 2,4-D, Mg/Al-LDH, Mg/Fe-LDH, Adsorption mechanisms, Loss control

## Abstract

Low efficiency and high surface runoff of 2,4-dichlorophenoxyacetic acid (2,4-D) from agricultural field threaten crop yield severely. Layered double hydroxides (LDH) have shown promising adsorption properties for 2,4-D. However, the comparison of two environmentally friendly LDHs (i.e. Mg/Al-LDH vs Mg/Fe-LDH) on adsorption of 2,4-D and corresponding intrinsic mechanisms are still unclear, and the studies on the leaching control of 2,4-D by LDHs in soil environment are particularly limited. In this study, Mg/Al-LDH and Mg/Fe-LDH were selected to conduct their adsorption kinetics experiment for 2,4-D combined with the characterization technology. The results showed that the adsorption capacity of Mg/Al-LDH and Mg/Fe-LDH for 2,4-D was up to 242 mg kg^−1^ and 64 mg kg^−1^, respectively, which were negatively correlated with pH. Adsorption mechanisms of both Mg/Al-LDH and Mg/Fe-LDH for 2,4-D are dominated by chemical adsorption, including electrostatic attraction and inner sphere complexation, but no interlayer adsorption mechanism. Mg/Al-LDH contains smaller metal ion radius, which provides greater surface charge density, resulting in greater electrostatic attraction and inner sphere complexation to 2,4-D compared to Mg/Fe-LDH. The greater adsorption capacity of Mg/Al-LDH for 2,4-D was driven by the higher adsorption energy (*E*_ads_) and lower electron density, as corroborated by density functional theory (DFT) calculation. The soil column experiment further verified that Mg/Al-LDH could control the loss of 2,4-D more effectively, and the leaching amount could be significantly reduced by 61.7%, while the effect of Mg/Fe-LDH was only 24.2%. This study provides theoretical guidance for screening more potential LDH types to solve the leaching loss of 2,4-D from soil and improve its effectiveness in agricultural production.

**Graphical Abstract:**

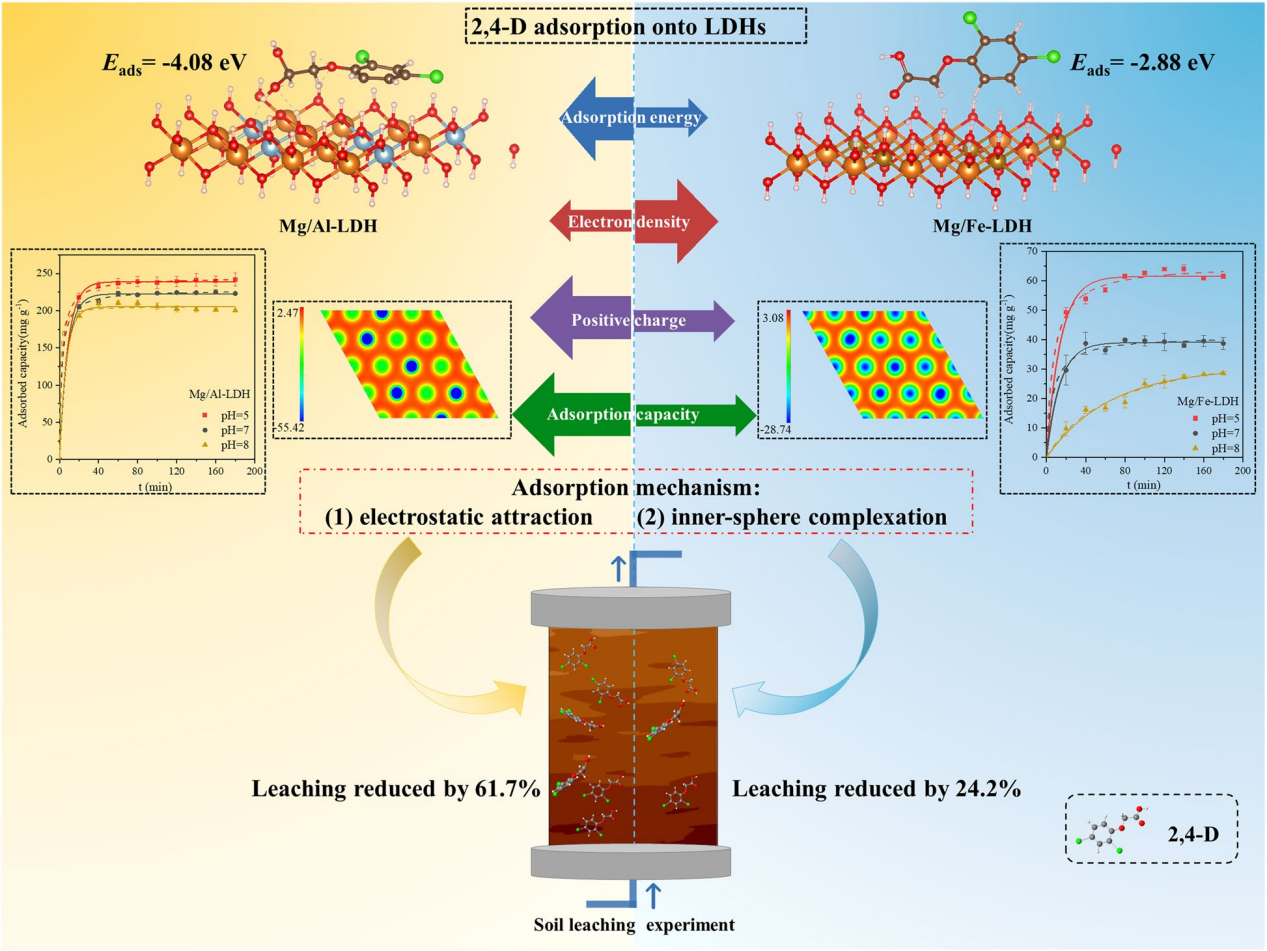

**Supplementary Information:**

The online version contains supplementary material available at 10.1007/s44307-024-00055-3.

## Introduction

2,4-dichlorophenoxyacetic acid (2,4-D), as a typical benzene carboxylic acid herbicide, is one of the highest usage amounts of herbicides worldwide (Li et al. 2011). However, 2,4-D is easy to leach and migrates into nearby water and groundwater, aggravating the pollution risk of water environment (Hall et al. 1993; Wood and Anthony 1997; Balinova and Mondesky 1999). In some areas of excessive pesticide application or heavy rainfall, the loss of 2,4-D from soil is as high as 91%, making it impossible for 2,4-D to reach the target weeds accurately and efficiently, thus reducing its biological availability and threatening crop yields (Shi et al. 2021). On the other hand, the irregular migration of 2,4-D in soil also poses a serious threat to soil ecological stability, so that soil microorganisms are disturbed and stressed, which is not conducive to soil nutrient cycling (Zabaloy et al. 2010; Oleszczuk et al. 2014). Therefore, reducing the migration and loss of 2,4-D in soil and improving its target efficiency have become a hot research spot.

At present, the removal methods of 2,4-D in water include O_3_ oxidation, fenton oxidation, electrochemical degradation and adsorption methods (Rodríguez et al. 2013; Conte et al. 2012; Souza et al. 2016; Liu et al. 2016). The O_3_ oxidation method has high energy consumption and operating costs (Rodríguez et al. 2013). The fenton oxidation method cannot treat alkaline wastewater (Conte et al. 2012), and electrochemical degradation is easily polluted by intermediate products, resulting in reduced current efficiency (Souza et al. 2016). In contrast, adsorption method can achieve the enrichment and separation of 2,4-D in water well, and the adsorbent can be reused repeatedly (Liu et al. 2016). In recent years, layered double hydroxides (LDHs) have attracted much attention because of their good adsorption properties, and have been widely used in various fields, especially agriculture. Certain types of LDHs have been found to adsorb 2,4-D in aqueous solutions, such as Mg-Zn-Al-LDH, Zn-Al-Cl-LDH, [Co-Al-Cl] LDH, Cu-Fe-NO_3_-LDH, Mg/Al-LDH and [Li-Al-Cl] LDH (Valente et al. 2009; Legrouri et al. 2005; Calisto et al. 2019; Nejati et al. 2013; Cardoso et al. 2006; Ragavan et al. 2006). LDHs consist of positively charged metallic layer and interlayer filled with exchangeable anions and water molecules (Zhang et al. 2002). Some of the bivalent metal cations on the layer are replaced by trivalent cations with similar radius, so that the layer is always positively charged, and anions are inserted between the layers to maintain the overall charge balance.

Mg/Al-LDH and Mg/Fe-LDH are frequently used as anion sustained-release agents because of their better environmental friendliness (Valente et al. 2009; Cardoso et al. 2006; Zhang et al. 2018; Hudcová et al. 2022; Gao et al. 2020; Jiang et al. 2019). Mg and Fe are both important trace elements of crops, and Al often exists in the form of hydroxide in the soil environment with pH ≥ 5, and does not produce toxic aluminum ion (Kanmegne et al. 2000). It has been found that Mg/Al-LDH has a stronger adsorption capacity for phosphate, but Mg/Fe-LDH has a greater adsorption potential for arsenate. This is due to the higher affinity of phosphate for aluminum and the stronger affinity of arsenate for iron (Caporale et al. 2011). On the other hand, compared with Mg/Al-LDH, Mg/Fe-LDH has lower Fe-OH bond energy and can remove the -OH of the laminate at a lower temperature (Zhang et al. 2002), which is more advantageous in the intercalation adsorption. However, the comparison between Mg/Al-LDH and Mg/Fe-LDH adsorption of 2, 4-D is lacking. Furthermore, current studies have mainly focused on the adsorption of 2,4-D by LDH in aqueous systems, there is little information about the effect of LDH application on the transport process of 2,4-D in complicated soil environments, which could better reflect the real application scenarios. Therefore, the adsorption capacity and intrinsic mechanism of Mg/Al-LDH and Mg/Fe-LDH to 2,4-D were compared in our study. The effect of both LDHs on leaching of 2,4-D from soil columns was further explored. These studies could provide scientific significance for improving the target effectiveness of pesticides and reducing environmental pollution.

## Materials and methods

### Preparation of Mg/Al-LDH and Mg/Fe-LDH

The chemical reagents used in the experiment were all analytical grade (AR). Mg/Al-LDH and Mg/Fe-LDH were synthesized by conventional co-precipitation method (Gao et al. 2018). 250 mL salt solution (1 M, Mg^2+^/Al^3+^ or Mg^2+^/Fe^3+^ mole ratio is 2:1) and alkali solution (2 M NaOH) were simultaneously introduced into a 2 L beaker containing 250 mL of NaCl. The pH was maintained at 10, and the resulting mixture was continuously stirred for a duration of 100 min under an inert nitrogen atmosphere. Subsequently, the mixtures were allowed to age for 2 h at room temperature (25 °C), followed by washing with deionized water until the effluent reached neutral pH. The products were then collected after being dried in an oven at 65 °C for 24 h. Finally, the dried materials were crushed and sieved in preparation for subsequent experiments.

### Adsorption kinetics experiment and computational method

200 mg L^−1^ of 2,4-D was prepared in 0.01 M CaCl_2_ solution, and the pH of the solution was adjusted to 5, 7 and 8, respectively. 0.05 g of Mg/Al LDH or Mg/Fe LDH was added to 300 mL of the above 2,4-D solution and the samples were taken at intervals. After sampling at each stage, 0.1 M HCl or NaOH was used to maintain a fixed pH level. An ultraviolet spectrophotometer determined the amount of 2,4-D in the supernatant. The pseudo-first-order model (1), pseudo-second-order model (2), and intra-particle diffusion model (3) were then used to fit the results of the determination (Annadurai et al. 2002; Ho 2006; Azizian 2004), with the relevant equations as follows:

1 $$\text{ln}\left({q}_{\text{e}}-{q}_{\text{t}}\right)=\text{ ln}{q}_{\text{e}}-{k}_{1}\text{t}$$

2 $${q}_{\text{t}}= {k}_{2}{{q}_{\text{e}}}^{2}\text{t}/(1+{k}_{2}{q}_{\text{e}}\text{t})$$

3 $${q}_{\text{t}}= {k}_{\text{d}}{\text{t}}^{\text{l}/2}+C$$

where *q*_t_ (mg g^−1^) and *q*_e_ (mg g^−1^) are 2,4-D adsorption amounts at time t and equilibrium time; *k*_1_ (min^−1^), *k*_2_ (min^−1^) and *k*_d_ (mg∙(g∙min^1/2^) ^−1^) are the rate constants for pseudo-first-order, pseudo-second-order models, and intra-particle diffusion model, respectively. *C* are the intercept of Intra-particle diffusion model.

Additionally, density functional theory (DFT) computations were carried out in the study using the Vienna Ab-initio Simulation Package (VASP). The microscopic interaction between LDHs and 2,4-D was studied by spin polarization DFT simulation using the Dmol3 program module. Supporting Information (SI) contained the DFT calculation specifics.

### Characterization

X-ray diffraction (XRD, PANalytical B.V., Holland) were employed to observe the crystallographic structure of Mg/Al-LDH and Mg/Fe-LDH with and without the adsorption of 2,4-D. Fourier transform infrared spectroscopy (FTIR, ThermoFisher Scientific, USA) and X-ray photoelectron spectroscopy (XPS, ThermoFisher Scientific, USA) were conducted to determine the difference in functional groups among the samples. The specific surface area and pore characteristics of the samples were performed by the Brunauer Emmett Teller (BET) based on N_2_ adsorption methods. Zeta potentials of LDHs and 2,4-D reacted LDHs under different pH gradients from 3 to 13 were measured using a Zetasizer (Malvern). Specific details of the assay were provided in the SI.

### Soil leaching experiment

The surface soil samples (1–20 cm) were collected from an agricultural field in Guangzhou, China in 2020. The soil contained 3.08% clay, 54.48% silt and 42.44% sand according to the hydrometer method (Chen 2010). The soil pH was 5.25 ± 0.05 and cation exchange capacity was 1.97 cmol L^−1^. Soils were mixed with 0.5% Mg/Al-LDH or Mg/Fe-LDH by shaking over 1 h vertically before column packing.

An opaque PVC leaching column (50 cm in length, 5 cm in inner diameter) was filled with glass wool and fine quartz sand. After leveling off the 50 g soil at the bottom of the column, full wetting is achieved by adding 50 mL 0.01 M CaCl_2_. 800 g soil with and without 0.5% LDH was packed into the PVC pipe column. Then, 400 mg 2,4-D (i.e. 500 mg kg^−1^ soil) was diluted with 1 mL 0.01 M CaCl_2_ solution and added to the upper of each soil column. 0.01 M CaCl_2_ solution was injected into the soil column at a speed of 25 mL per day for 12 days, and the leachate was collected every two days. The 2,4-D in the leachate was determined by ultraviolet spectrophotometer at 284 nm (Gordon and Beroza 1952). After leaching experiment, the soil samples from the columns were excavated in 10 cm increments. Soil of each layer was freeze-dried, grind and fully mixed. 1 g soil was mixed with 20 mL methanol, ultrasonicated at room temperature for 30 min, and then centrifugated. The 10 mL of methanol was added to the precipitate and repeated the steps above three times and all the supernatants were collected and concentrated into 1 mL with N_2_ before being measured with high-performance liquid chromatography (HPLC) to obtain the concentration of 2,4-D.

### Statistical analysis

The significance analysis was carried out with IBM SPSS statistics 25. One-way ANOVA analyses were conducted to determine the differences of 2,4-D in soil leaching solution and in soil column residue before and after the addition of 0.5% Mg/Al-LDH or Mg/Fe-LDH. Tukey's honestly significant differences (HSD) test was used to compute differences between mean values at a significance level of 0.05. The figures were created using OriginPro 2021 (OriginLab Corp., Northampton, MA, USA).

## Results and discussion

### The synthesis of Mg/Al-LDH and Mg/Fe-LDH

**Fig. 1:**
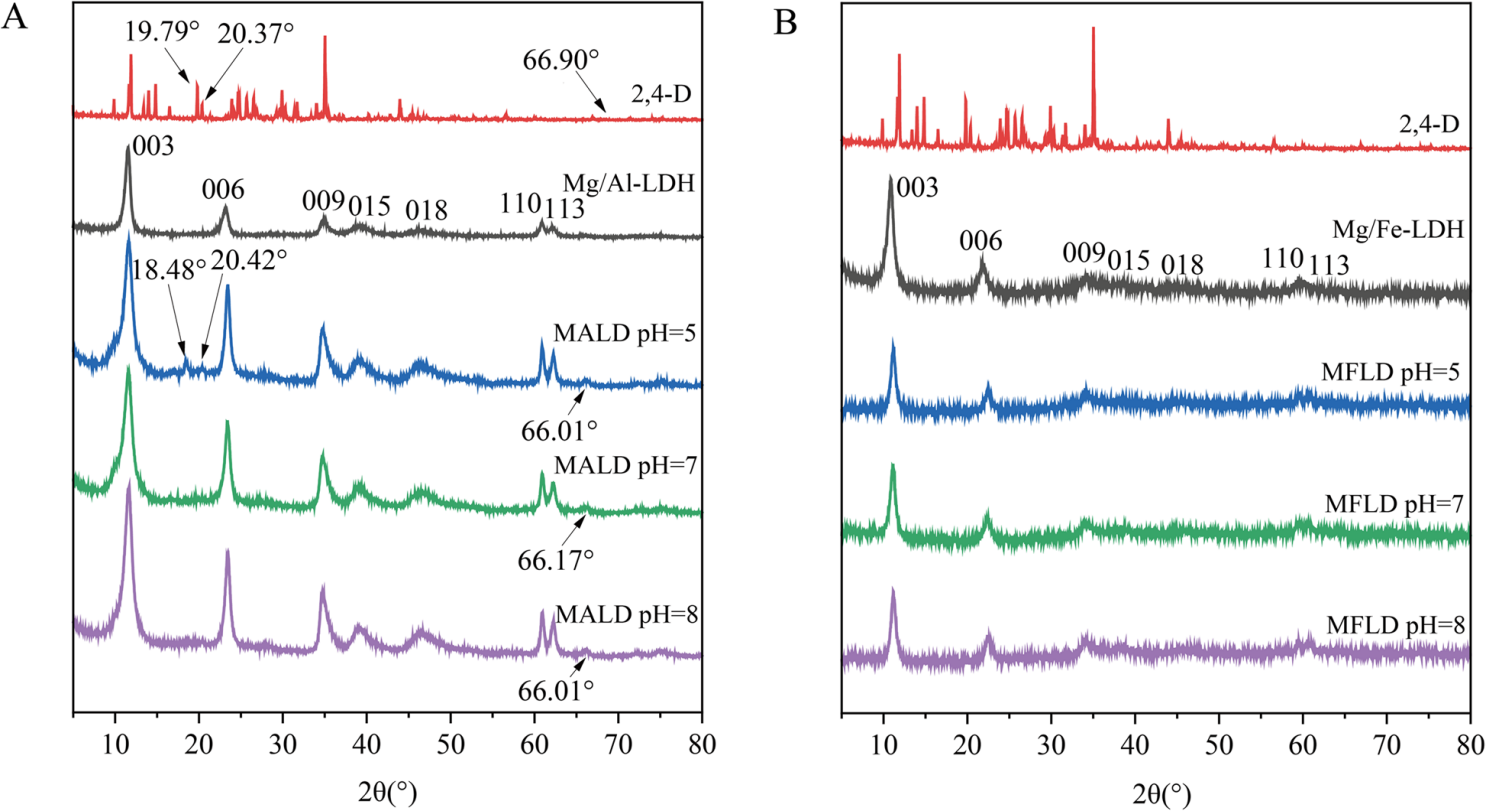
XRD results of Mg/Al-LDH and Mg/Fe-LDH before and after adsorption of 2,4-D at pH = 5, 7, and 8. (MALD: Mg/Al-LDH after adsorption of 2,4-D; MFLD: Mg/Fe-LDH after adsorption of 2,4-D)

**Fig. 2:**
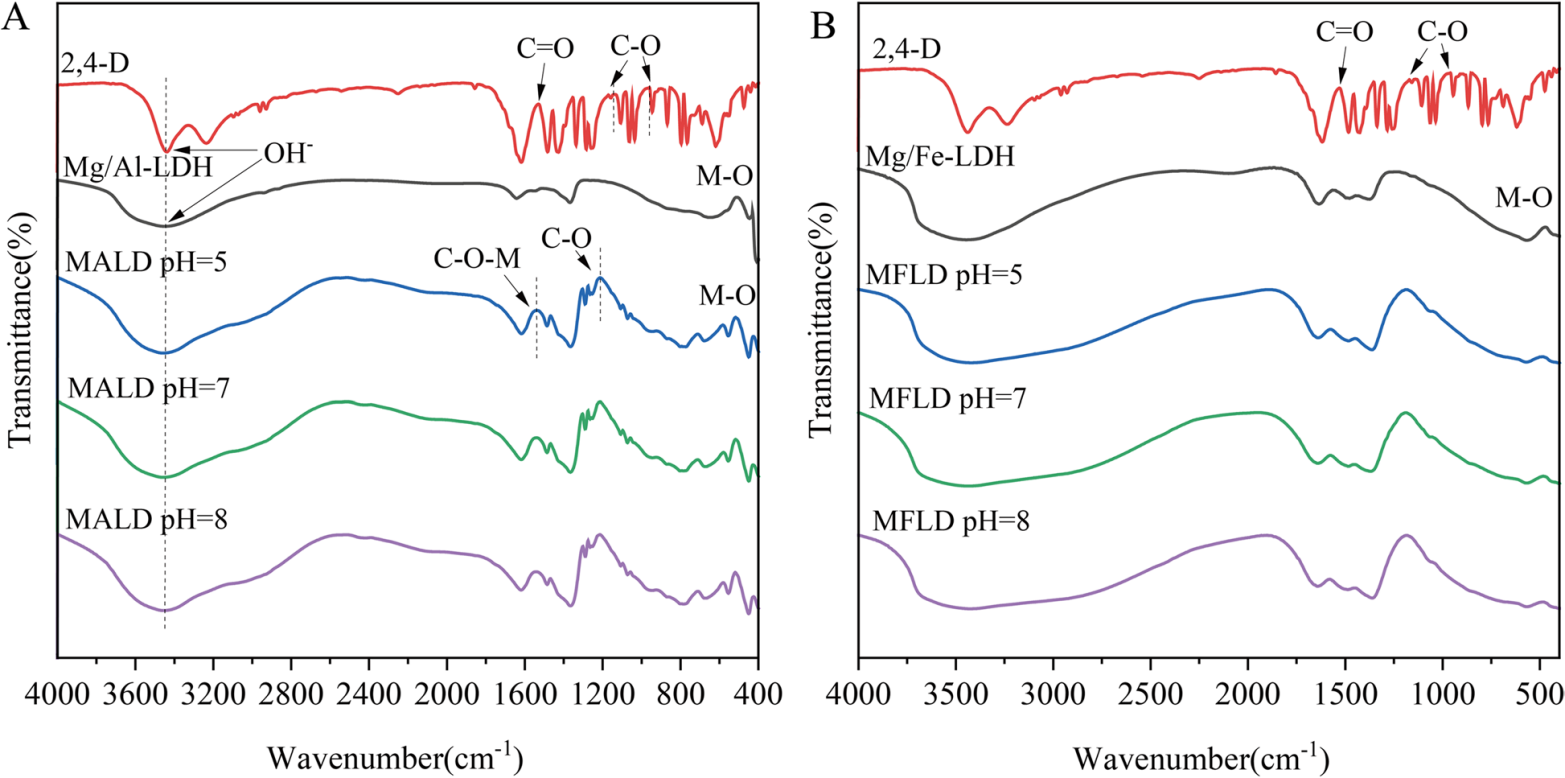
FTIR (**A** and **B**) results of 2,4-D, Mg/Al-LDH and Mg/Fe-LDH before and after adsorption of 2,4-D at pH = 5, 7, and 8. (MALD: Mg/Al-LDH after adsorption of 2,4-D; MFLD: Mg/Fe-LDH after adsorption of 2,4-D)

The effective synthesis of Mg/Al-LDH and Mg/Fe-LDH was demonstrated by the XRD (Fig. 1) and FTIR (Fig. 2) data. Peaks (003), (006), (009), (015), (018), (110) and (113) (Fig. 1A and B) were present in both the synthesized Mg/Al-LDH and Mg/Fe-LDH, and they were consistent with the standard LDH model diagram (Kang et al. 2013; Ahmed and Gasser 2012). In addition, in contrast to Mg/Fe-LDH, the peaks of Mg/Al-LDH were characterized by narrower half-peak width, sharper peak shape and fewer impurity peaks, indicating higher crystallinity (Singh and Sudip 2022). The FTIR results revealed that the absorption peaks of Mg/Al-LDH and Mg/Fe-LDH at 3400–3500 cm^−1^ and 3200–3800 cm^−1^, respectively (Fig. 2), corresponding to the stretching vibration of -OH in the laminar (Zhang et al. 2019). The acromion at 1300–1700 cm^−1^ for Mg/Al-LDH (Fig. 2A) and 1300–1600 cm^−1^ for Mg/Fe-LDH (Fig. 2B) may be attributed to the O–H bending vibration of interlayer water. The absorption peaks below 500 cm^−1^ were ascribed to the lattice vibrations of Mg-O, Al-O and Fe–O between the laminates (Caporale et al. 2011). These results indicated the successful synthesis of Mg/Al-LDH and Mg/Fe-LDH. The absorption peak of Mg/Al-LDH at 650 cm^−1^ was caused by translational vibration of -OH and H_2_O. The corresponding -OH stretching region and metal band in the Mg/Fe-LDH infrared map were wide, which also indicated that Mg/Fe-LDH had a low crystallinity of the particles (Caporale et al. 2011).

### Comparative adsorption of Mg/Al-LDH and Mg/Fe-LDH to 2,4-D at different pH

**Fig. 3:**
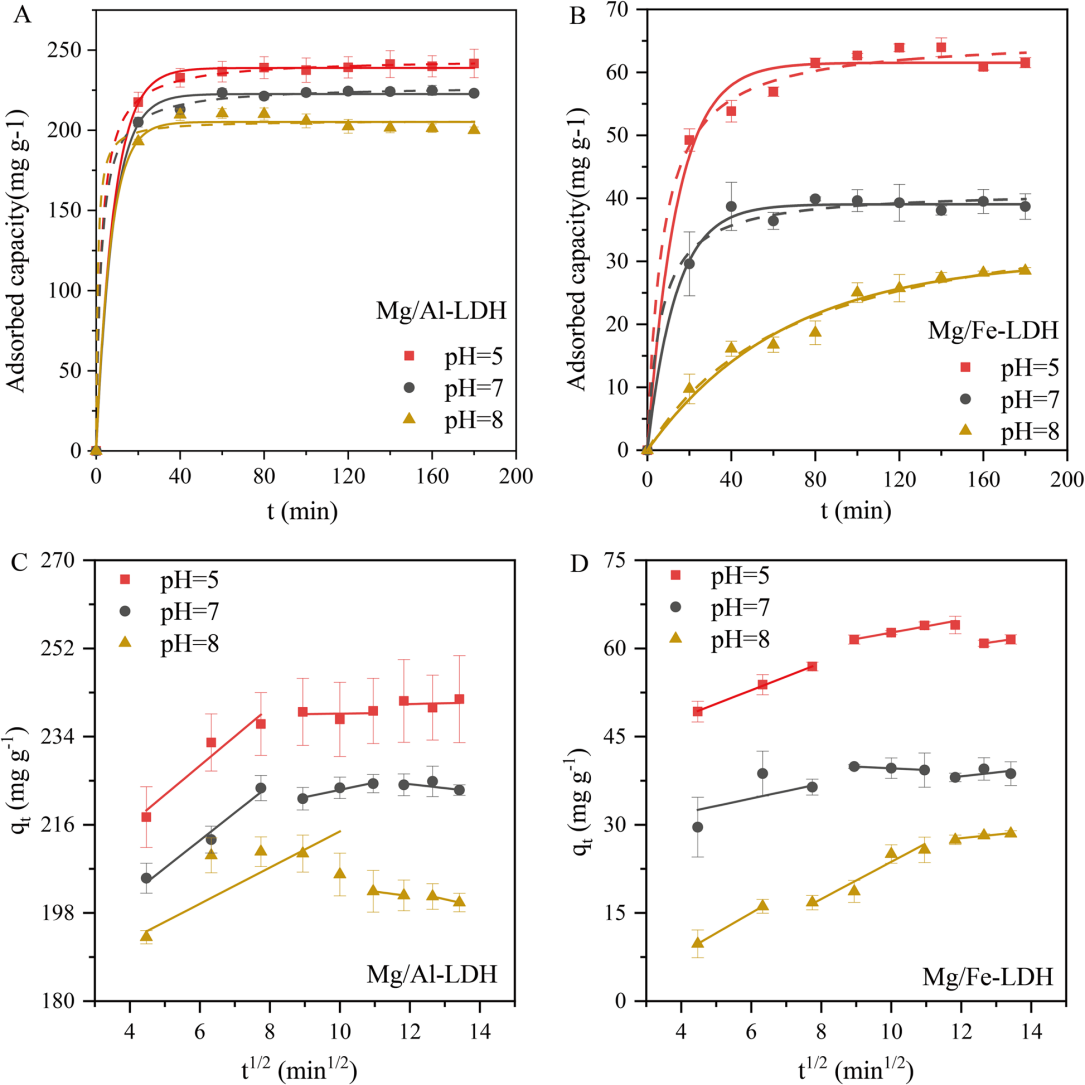
Pseudo-first-order model (**A**, **B**: solid line), pseudo-second-order model (**A**, **B**: dashed line) and intra-particle diffusion model (**C**, **D**: solid line) for the adsorption of 2,4-D by Mg/Al-LDH (**A**, **C**) and Mg/Fe-LDH (**B**, **D**) at pH = 5, 7, and 8

The adsorption kinetic results of two LDHs on 2,4-D adsorption at pH = 5, 7, and 8 were shown in Fig. 3A and B. Both LDHs exhibited obvious pH responses, showing a trend of rapid adsorption at the initial stage but slow and stable adsorption at the later stage. LDHs provided more adsorption sites in the initial stage of the adsorption reaction with a faster adsorption rate. The adsorption sites on the surface of LDH decreased gradually during the adsorption reaction, resulting in the decrease of the adsorption rate until reaching adsorption equilibrium (Singh and Sudip 2022). The maximum adsorption capacity of Mg/Al-LDH for 2,4-D was 240 mg g^−1^, 220 mg g^−1^ and 200 mg g^−1^ at pH = 5, 7, and 8, respectively. In contrast, the maximum adsorption capacity of Mg/Fe-LDH for 2,4-D was 65 mg g^−1^, 40 mg g^−1^ and 28 mg g^−1^ at pH = 5, 7, and 8, respectively. It indicated that the 2,4-D adsorption capacity of Mg/Al-LDH was obviously higher than that of Mg/Fe-LDH, and the maximum adsorption capacity of both LDHs for 2,4-D was negatively correlated with pH. The increase of negative charge binding sites on the surface of LDH by protonation at lower pH improved the adsorption capacity of both LDHs to 2,4-D (Sarkar et al. 2010; Zhang et al. 2012). Pseudo-first-order dynamic and pseudo-second-order dynamic models were selected to fit the experimental data (Table 1) (Tan and Hameed 2017; Su et al. 2013), and both performed well in predicting 2,4-D experimental data (*R*^2^ ≈ 0.99). It indicated that the adsorption process is probably controlled by both physical and chemical processes, and the adsorption process was the combined action of intermolecular van der Waals forces and strong chemical bonding forces (Xie et al. 2018). Since pseudo-first-order and pseudo-second-order equations can not identify the diffusion mechanism, the intraparticle diffusion model was also tested (Fig. 3C and D). Generally, the adsorption process consists of the following four steps: (1) bulk diffusion, (2) film diffusion, (3) intra-particle diffusion, and (4) solute adsorption (Albadarin et al. 2012). The intra-particle diffusion model analysis showed that the adsorption processes of 2,4-D by both Mg/Al-LDH and Mg/Fe-LDH were divided into three linear segments (Fig. 3C and D), and the parameters of these models were shown in Table S2. Both plots of Mg/Al-LDH and Mg/Fe-LDH adsorbed with 2,4-D presented multilinearity, indicating that the absorption of 2,4-D was a heterogeneous process, including external surface sorption and intraparticle diffusion, dominated by multiple mechanisms (Radnia 2011; Wang et al. 2024). The slope *K*_d1_ of the first linear phase was the largest, indicating that 2,4-D crossed the liquid film and reached the adsorbent surface rapidly (film diffusion). The *K*_d1_ of Mg/Al-LDH (5.97) was larger than that of Mg/Fe-LDH (2.32), which implies that 2,4-D can reach the surface of Mg/Al-LDH faster. It may be the key factor of the difference in adsorption amount between Mg/Al-LDH and Mg/Fe-LDH. The second phase reflected that 2,4-D continued to move into the adsorbed interior (intra-particle diffusion). The slope became flat, suggesting that intra-particle diffusion was the rate-limiting step for adsorption (Yang et al. 2021). The final stage was solute adsorption when adsorption reached dynamic equilibrium.

**Table 1:** Adsorption kinetic fitting parameters for adsorption experiments

Adsorbent	Pseudo-first-order dynamic equation			Pseudo-second-order dynamic equation		
	*q*_e_/(mg∙g^−1^)	*k*/min^−1^	*R*^2^	*q*_e_/(mg∙g^−1^)	*k*/min^−1^	*R*^2^
MALD pH = 5	238.88	0.12	0.99	244.79	0.00	0.99
MALD pH = 7	222.61	0.12	0.99	227.94	0.00	0.99
MALD pH = 8	205.22	0.14	0.99	206.22	0.00	0.99
MFLD pH = 5	61.52	0.07	0.98	65.63	0.00	0.99
MFLD pH = 7	39.06	0.07	0.99	41.28	0.00	0.99
MFLD pH = 8	30.40	0.02	0.97	40.37	3.48	0.98

*MALD* Mg/Al-LDH after adsorption of 2,4-D, *MFLD* Mg/Fe-LDH after adsorption of 2,4-D

**Fig. 4:**
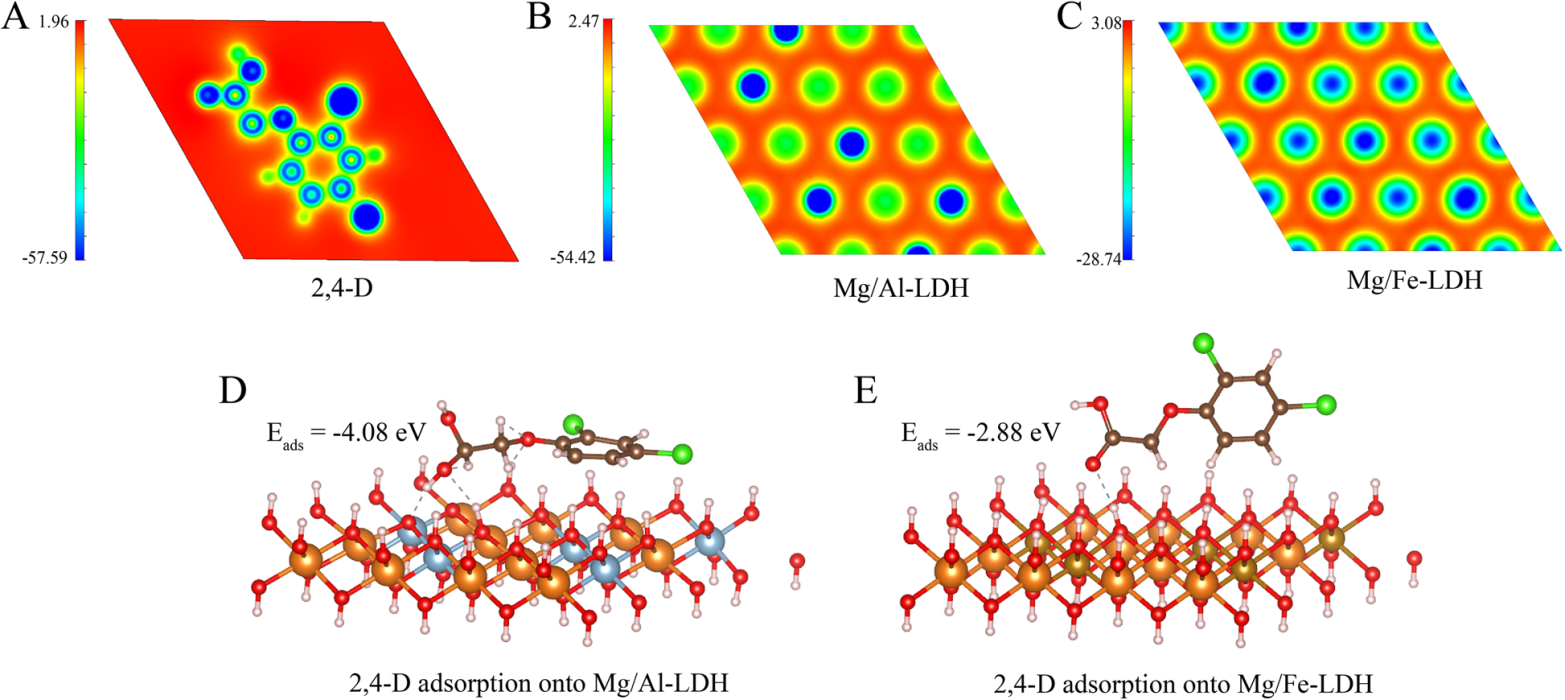
The electrostatic potential distribution (ESP) of (**A**) 2,4-D, (**B**) Mg/Al-LDH, and (**C**) Mg/Fe-LDH; the optimized configurations and adsorption energies (*E*_ads_) of 2,4-D adsorption onto (**D**) Mg/Al-LDH and (**E**) Mg/Fe-LDH

The XRD results showed that the absorption peaks of Mg/Al-LDH adsorbed with 2,4-D (pH = 5) at 18.48° and 20.42° corresponded to the absorption peaks of 2,4-D, indicating that 2,4-D was loaded onto Mg/Al-LDH successfully. However, this phenomenon was not manifested at Mg/Al-LDH adsorbed with 2,4-D at pH = 7 and 8, which may be due to less adsorption of 2,4-D onto Mg/Al-LDH at pH = 7 and 8. In contrast, there was no significant difference in the absorption peaks between Mg/Fe-LDH and Mg/Fe-LDH adsorbed 2,4-D at pH = 5, 7 and 8, probably also due to the small adsorption capacity of Mg/Fe-LDH to 2,4-D. Mg/Fe-LDH had a wider peak of d_003_ compared to Mg/Al-LDH (Mg/Fe-LDH: 0.82 nm > Mg/Al-LDH: 0.76 nm). This may be since the ionic radius of the Fe^3+^ (0.065 nm) is larger than that of Al^3+^ (0.051 nm) (Zhang et al. 2002; Lin et al. 2014). Furthermore, BET results showed that Mg/Fe-LDH could provide a more specific surface area than Mg/Al-LDH (Table 2), which showed that Mg/Fe-LDH had a better structure on physical adsorption than Mg/Al-LDH. However, the adsorption capacity of Mg/Fe-LDH to 2,4-D was lower in fact, suggesting that the adsorption of Mg/Fe-LDH to 2,4-D may not be relied on physical adsorption, and the adsorption was dominated by chemical adsorption. This conclusion was also supported by the results of the DFT calculation. Figure 4D and E showed the optimized configuration of Mg/Al-LDH and Mg/Fe-LDH adsorp 2,4-D and the corresponding adsorption energies (*E*_ads_). Higher values of adsorption energy indicate the easier adsorption of 2,4-D (Zhang et al. 2024). The *E*_ads_ of both Mg/Al-LDH and Mg/Fe-LDH were less than −0.5 eV, indicating that the adsorption process was dominated by chemical adsorption (Cai et al. 2022). Additionally, the order of energies absolute values of 2,4-D adsorption onto Mg/Al-LDH and Mg/Fe-LDH were as follows: Mg/Al-LDH (*E*_ads_ = −4.08 eV) > Mg/Fe-LDH (*E*_ads_ = −2.88 eV), suggesting that the greater adsorption capacity of Mg/Al-LDH for 2,4-D was driven by the higher *E*_ads_. In addition, Table 2 showed that the BET surface area, pore volume and pore diameter of both two LDH for 2,4-D were the smallest at pH = 7. it may mean that the physical adsorption of both two LDHs was the smallest at pH = 7. The maximum adsorption capacity of both Mg/Al-LDH and Mg/Fe-LDH for 2,4-D is negatively correlated with pH (Table 1). It illustrated that the physical adsorption capacity of LDHs for 2,4-D was not correspond to the total adsorption capacity one by one, suggesting that the adsorption of LDHs to 2,4-D may not be relied on physical adsorption, and the adsorption was dominated by chemical adsorption at pH = 7.

**Table 2:** The BET surface area, pore volume and pore diameter of Mg/Al-LDH and Mg/Fe-LDH before and after adsorption of 2,4-D at pH = 5, 7, and 8

Materials	BET Surface Area (m^2^ g^−1^)	Pore volume (cm^3^ g^−1^)	Pore diameter (nm)
Mg/Al-LDH	9.4706	0.037259	15.7367
MALD pH = 5	70.4096	0.278173	15.8032
MALD pH = 7	41.4943	0.138687	13.3693
MALD pH = 8	59.5976	0.205894	13.8189
Mg/Fe-LDH	71.7215	0.057362	3.1992
MFLD pH = 5	101.1087	0.178685	7.069
MFLD pH = 7	52.902	0.053203	4.0228
MFLD pH = 8	99.2776	0.194226	7.8256

*MALD* Mg/Al-LDH after adsorption of 2,4-D, *MFLD* Mg/Fe-LDH after adsorption of 2,4-D

### Comparative study on electrostatic attraction of Mg/Al-LDH and Mg/Fe-LDH to 2,4-D

The adsorption mechanisms of Mg/Al-LDH and Mg/Fe-LDH for 2,4-D were further explored. The FTIR results showed that electrostatic adsorption exists between Mg/Al-LDH and 2,4-D (Fig. 2A). Compared the intensity, shape and position changes of FTIR peaks of Mg/Al-LDH before and after adsorption of 2,4-D, we found that Mg/Al-LDH with the adsorption of 2,4-D had the corresponding adsorption peaks of 2,4-D (i.e. 1487 cm^−1^, 1290 cm^−1^, 1254 cm^−1^, 1109 cm^−1^, 1072 cm^−1^, 793 cm^−1^ and 555 cm^−1^). It implied that 2,4-D was loaded onto Mg/Al-LDH successfully. The C-O-M vibration of the -COOH at 1400–1600 cm^−1^ for Mg/Al-LDH after adsorption of 2,4-D moved to a higher wave, indicating that hydrogen bond was formed or proton transfer occurred (Chang and Lv 2009). Generally, the formation of hydrogen bond is accompanied by the shift of absorption peak to the lower wave number (red shift), which was caused by the decrease of the bond force constant between hydrogen atoms and their connected atoms and a lower vibration frequency (Kanezaki and Katoh 2011). Nevertheless, the vibration of -OH at 3430 cm-1 and the O–H bending vibration of interlayer water at 1598 cm-1 and 1725 cm-1 had no movement, which indicated that no hydrogen bond occurred during the adsorption of 2,4-D onto Mg/Al-LDH. It implied that the movement of C-O-M vibration peak after adsorption of 2,4-D by Mg/Al-LDH may not rely on hydrogen bond but on proton transfer. Thus, the adsorption mechanism of Mg/Al-LDH to 2,4-D included the electrostatic attraction caused by proton transfer (Chang and Lv 2009; Zhang et al. 2015). It has to be mentioned that the FTIR peaks of Mg/Fe-LDH after adsorption of 2,4-D at pH = 5, 7, and 8 had no obvious change, probably also due to the small adsorption capacity of Mg/Fe-LDH to 2,4-D and could not be detected by FTIR.

**Fig. 5:**
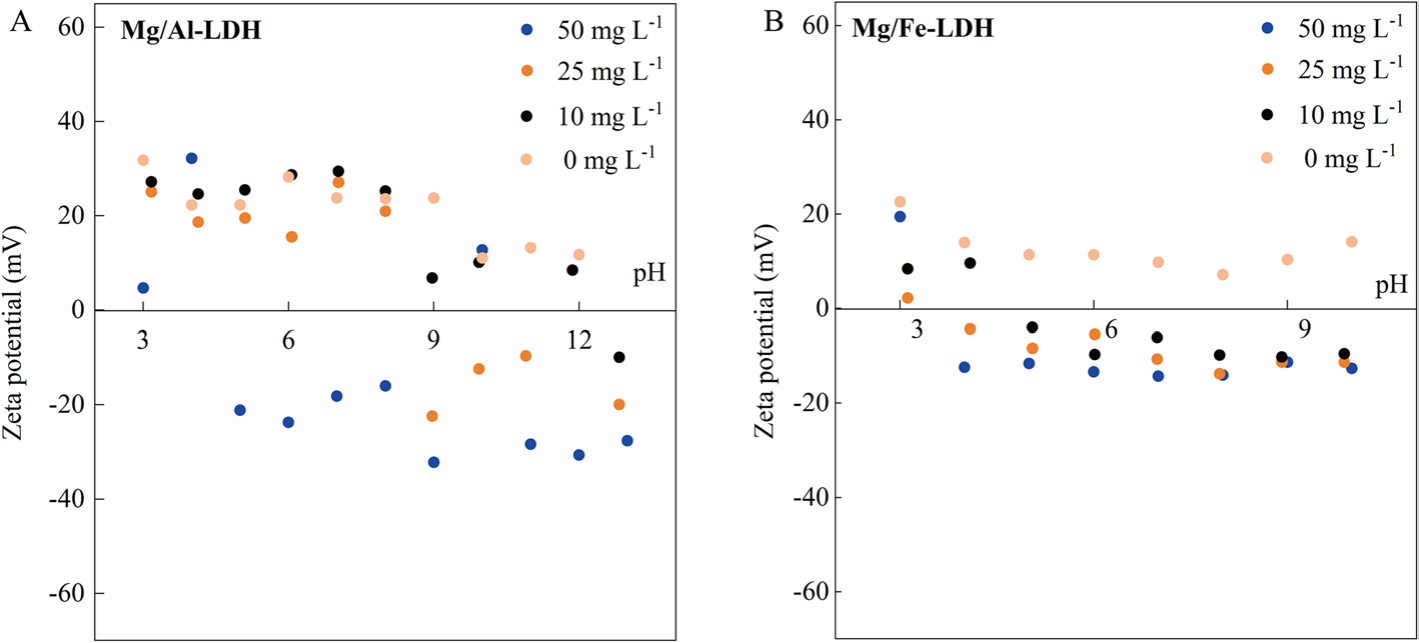
Zeta potential results of Mg/AL-LDH (**A**) and Mg/Fe-LDH (**B**) before and after adsorption of 2,4-D at pH = 5, 7, and 8. (The concentration gradients of 2,4-D were 50 mg L^−1^, 25 mg L^−1^, 10 mg L^−1^, 0 mg L.^−1^)

The adsorption mechanisms of Mg/Al-LDH and Mg/Fe-LDH for 2,4-D were further explored by zeta potential. According to the results of zeta potential (Fig. 5), both adsorption mechanisms of Mg/Al-LDH and Mg/Fe-LDH to 2,4-D include outer electrostatic attraction. In Fig. 5A and B, two types of LDHs were positively charged in the background solution of NaCl. With the increase of pH, OH^−^ in the solution gradually increased, resulting in the increase of the -OH on the active surface of LDH and the decrease of zeta potential. In addition, the pKa of 2,4-D is 2.73 (Nelson and Faust 1969), so it exists in anionic form at pH > 2.73, indicating that there was electrostatic attraction between LDHs and 2,4-D. Furthermore, Mg/Al-LDH in the background solution of NaCl has more positive charge than Mg/Fe-LDH based on the zeta potential results (Mg/Al-LDH: 12.2 ~ 32.2 mV, Mg/Al-LDH: 8.3 ~ 19.5 mV) (Fig. 5A and B). This may be since the ionic radius of the Al^3+^ (0.051 nm) is smaller than that of Fe^3+^ (0.065 nm) (Zhang et al. 2002; Lin et al. 2014), resulting in higher charge density for Mg/Al-LDH than the case of Mg/Fe-LDH. To further elucidate the extent of electrostatic adsorption in the contribution of Mg/Al-LDH and Mg/Fe-LDH to 2,4-D adsorption, the electrostatic potential distribution (ESP) of 2,4-D, Mg/Al-LDH and Mg/Fe-LDH were carried out. The red fields and blue areas of ESP for 2,4-D described the high electrons density and low electrons density, respectively (Fig. 4A). The edges of the 2,4-D molecule have a high electron density, which can generate abundant negative charges, thus allowing Mg/Al-LDH and Mg/Fe-LDH to adsorb 2,4-D by electrostatic attraction. Notably, compared to Mg/Fe-LDH, Mg/Al-LDH has lower electron density (Fig. 4B vs C) and carries more positive charge, thus the effect of electrostatic attraction with 2,4-D is stronger. These results indicated that the electrostatic attraction capacity of Mg/Al-LDH was higher than Mg/Fe-LDH, which was consistent with the adsorption kinetics and FTIR results.

### Comparative study on inner sphere complexation of Mg/Al-LDH and Mg/Fe-LDH to 2,4-D

**Fig. 6:**
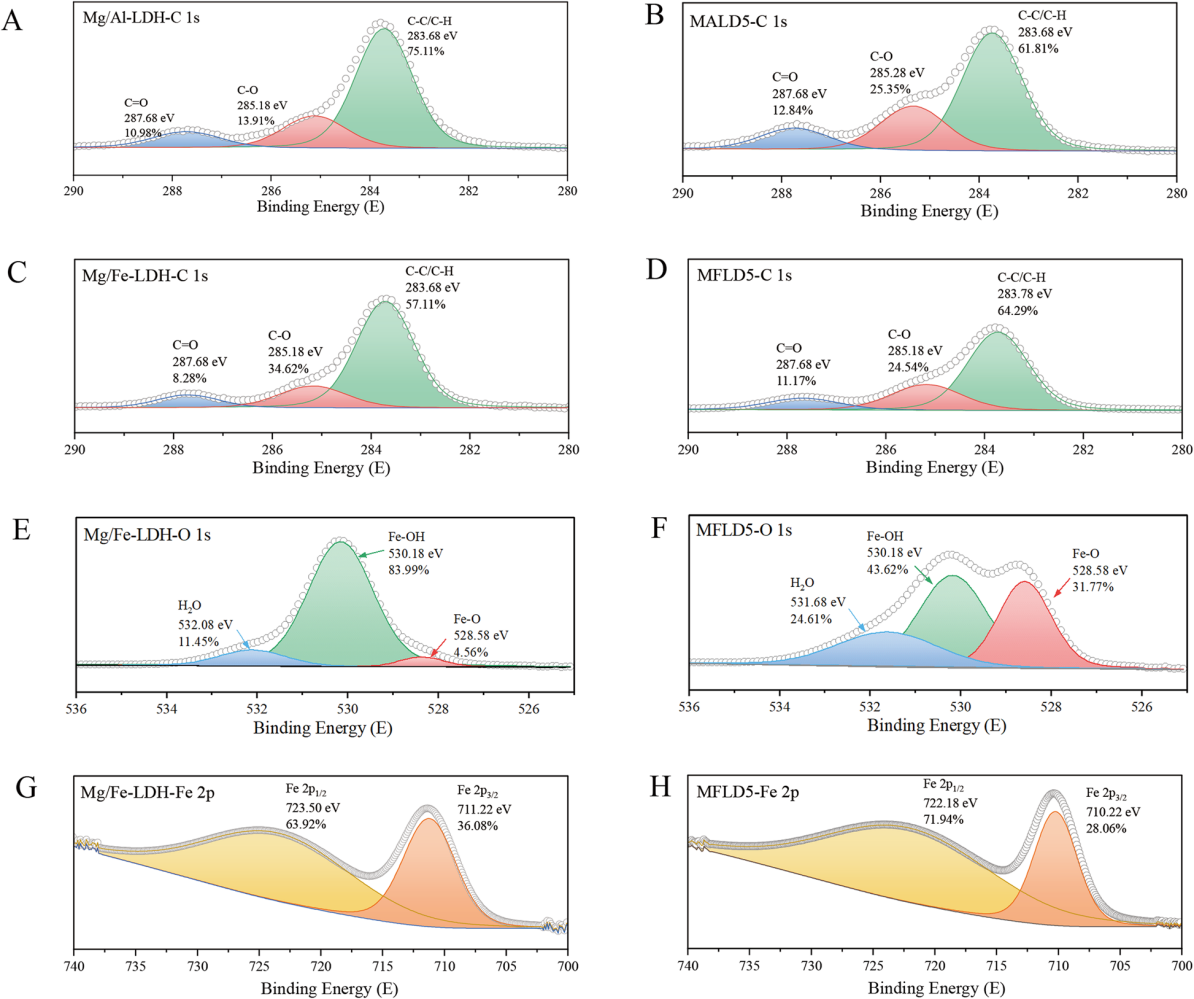
XPS results of Mg/Al-LDH and Mg/Fe-LDH before and after adsorption of 2,4-D at pH = 5. **A** and **B** C 1 s spectra Mg/Al-LDH before and after adsorption of 2,4-D; (**C** and **D**) C 1 s spectra of Mg/Fe-LDH before and after adsorption of 2,4-D; (**E** and **F**) Fe O1s spectra of Mg/Fe-LDH before and after adsorption of 2,4-D. **G** and **H** Fe 2p spectra of Mg/Fe-LDH before and after adsorption of 2,4-D (MALD: Mg/Al-LDH after adsorption of 2,4-D; MFLD: Mg/Fe-LDH after adsorption of 2,4-D)

Both adsorption mechanisms of Mg/Al-LDH and Mg/Fe-LDH to 2,4-D include inner sphere complexation based on the results of XPS and zeta potential. XPS spectrum analysis (Fig. S1) demonstrated that peaks of Mg 1 s, C 1 s, O 1 s, and Cl 2p emerged in the spectra of both Mg/Al-LDH and Mg/Fe-LDH. Meanwhile, the peaks of Al 2p and Fe 2p were respectively presented in the spectra of Mg/Al-LDH and Mg/Fe-LDH. Combined with the percentage content of each element measured by XPS full spectrum analysis in Table S1, it showed that the relative content of Cl element in Mg/Al-LDH after adsorbing 2,4-D (pH = 5, 7, and 8) was higher than Mg/Al-LDH distinctly, and had an increasing trend with the decrease of pH. This indicated that Mg/Al-LDH adsorbed 2,4-D successfully, and the adsorption capacity increased with decreasing pH. The result was consistent with XRD, FTIR and adsorption kinetics analysis. Compared the C 1 s energy spectrum of Mg/Al-LDH (Fig. 6A and B) with that of Mg/Fe-LDH (Fig. 6C and D), the relative content of C ls in Mg/Fe-LDH after adsorbing 2,4-D (pH = 5, 7, and 8) had no obvious change, showing lower adsorption capacity of Mg/Fe-LDH to 2,4-D. After adsorption of 2,4-D at pH = 5, 7 and 8, the content of C-O in the C 1 s energy spectrum of Mg/Al-LDH increased significantly, which further confirmed the successful loading of 2,4-D onto LDHs. Figure 6E and F show the O 1 s spectra of Mg/Fe-LDH with 2,4-D adsorption and without 2,4-D adsorption, respectively.

The O 1 s spectrums of Mg/Fe-LDH with and without the adsorption of 2,4-D were shown in Fig. 6E and F, respectively. Specifically, the photoelectron peaks at 532.08, 530.18, and 528.58 eV in the O 1 s spectra correspond to H_2_O, metal hydroxides M–OH, and metal oxide M–O (M: Fe), as reported by Yang et al. (2019). After adsorption of 2,4-D, the proportion of Fe–OH area decreased from 83.99% to 43.62%, and the proportion of Fe–O area increased from 4.56% to 31.77% for Mg/Fe-LDH. The decrease of Fe–OH indicated that –OH groups are likely to be occupied by 2,4-D molecules, and thus the inner-sphere complex occurred (Wang et al. 2015). Furthermore, the alterations can also be observed in Mg/Fe-LDH Fe 2p spectra with and without the adsorption of 2,4-D. The peak position of Fe 2p for Mg/Fe-LDH shifted toward low binding energy after absorbing 2,4-D, which proved the change of Fe − OH. It indicated that Mg/Fe-LDH to 2,4-D also included inner sphere complexation mechanisms.

The comparison of inner sphere complexation between Mg/Al-LDH and Mg/Fe-LDH for 2,4-D was further explored by zeta potential characterization. In Fig. 5A and B, the isoelectric point (IEP) of Mg/Al-LDH was ~ 12 when the concentration of 2,4-D was 10 mg L^−1^. The IEP of Mg/Al-LDH dropped to ~ 9 when the concentration of 2,4-D increased to 25 mg L^−1^, indicating that the inner-sphere complex occurs between Mg/Al-LDH and 2,4-D (Hunter 1981). Similarly, the IEP of Mg/Fe-LDH was > 4 when the concentration of 2,4-D was 10 mg L^−1^, and the IEP decreased to < 4 when the concentration of 2,4-D increased to 25 mg L^−1^. This was due to the fact that after the adsorption of 2,4-D, the -COOH of 2,4-D replaced the -OH of LDHs to form a strong coordination bond through ligand exchange reaction, and thus the inner-sphere complex occurred (Wang et al. 2022). The charge dispersion of Mg/Al-LDH with the adsorption of 2,4-D was found to be superior than that of Mg/Fe-LDH (Fig. 5A and B). Specifically, the potential range of Mg/Al-LDH during the adsorption process was identified as −30.6 ~ 32.2 mV, while for Mg/Fe-LDH, it was recorded at −12.6 ~ 19.5 mV. This observation suggested that Mg/Al-LDH had better dispersion in the aqueous media, thereby provided more abundant surface hydroxyl groups (Poemomo and Xu 2009). Consequently, there was a higher involvement of 2,4-D in the inner sphere adsorption onto Mg/Al-LDH as opposed to Mg/Fe-LDH.

Additionally, compared the XRD characteristic peak (d_003_) of Mg/Al-LDH (d_003_ = 0.760 nm) with that after the adsorption of 2,4-D at pH = 5, 7, and 8 (d_003_ = 0.775, 0.776, and 0.776 nm) (Fig. 1A), there was no significant difference in the layer spacing, indicating that the adsorption of 2,4-D onto Mg/Al-LDH was not dominated by interlayer ion exchange. Furthermore, the d_003_ layer spacing of Mg/Fe-LDH before and after adsorption of 2,4-D at pH = 5, 7, and 8 remained approximately 0.82 nm, further indicating no intercalation mechanism for the adsorption 2,4-D by Mg/Fe-LDH (Fig. 1B). Notably, the basal spacing of Cu/Fe-NO_3_ LDHs remained unchanged after the adsorption of 2,4-D (Nejati et al. 2013), which aligns with our experimental findings. Conversely, it has been observed that 2,4-D was intercalated into Zn/Al-Cl LDHs through ionic exchange when the concentration of 2,4-D exceeded 0.4 mmol L^−1^ (Legrouri et al. 2005). These findings suggested that the interlayer ionic exchange in LDHs was significantly influenced by varying experimental conditions.

### Comparative study of Mg/Al-LDH and Mg/Fe-LDH on loss control of 2,4-D from soil column

**Fig. 7:**
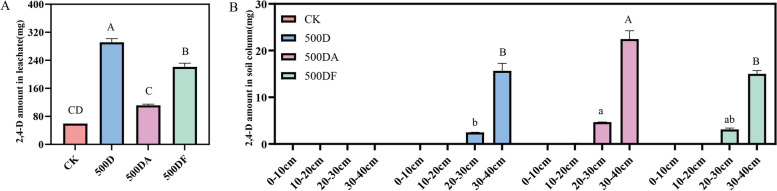
2,4-D amounts in soil leaching solution (**A**) and in soil column residue (**B**) before and after addition of 0.5% Mg/Al-LDH and Mg/Fe-LDH. (500D: 500 mg kg^−1^ 2,4-D treatment, 500DA: 500 mg kg^−1^ 2,4-D and 0.5% Mg/Al-LDH treatment, 500DF: 500 mg kg.^−1^ 2,4-D and 0.5% Mg/Fe-LDH treatment, One-way ANOVA analyses were conducted to determine the differences of 2,4-D in soil leaching solution and in soil column residue before and after addition of 0.5% Mg/Al-LDH and Mg/Fe-LDH at a significance level of 0.05)

The soil leaching experiment showed that the addition of Mg/Al-LDH and Mg/Fe-LDH reduced the leaching amount of 2,4-D from soil column significantly, especially for Mg/Al-LDH. The leaching rate of 2,4-D from soil column without the addition of LDHs was 75%, showing an extremely serious leaching risk of 2,4-D from soil. Interestingly, the leaching of 2,4-D was reduced by 61.7% and 24.2% (Table S2), respectively, after the addition of 0.5% Mg/Al-LDH and Mg/Fe-LDH in soil column (Fig. 7A). The results showed that Mg/Al-LDH and Mg/Fe-LDH had excellent loss control for 2,4-D in soil environment, especially for Mg/Al-LDH. Further analysis of soil columns with different depths after the transport of 2,4-D showed that 2,4-D was not detected in 0–20 cm soil layer in the case of with and without the addition of LDHs (Fig. 7B). The addition of Mg/Al-LDH promoted the accumulation of 2,4-D in 20–40 cm soil layer, and the residual amount of 2,4-D in 20–30 cm and 30–40 cm increased by 87.06% and 43.40% (Table S2), respectively. These results indicated that Mg/Al-LDH could control the loss of 2,4-D significantly in deeper soil (i.e. > 20 cm). This was since that 2,4-D of topsoil was leaching more easily into deeper soil along with a more intense CaCl_2_ solution wash. In contrast, there was no significant difference in the loss control of 2,4-D before and after the addition of Mg/Fe-LDH. Respectively, the residual amount of 2,4-D in 30–40 cm was 15.7 mg kg^−1^ and 15.0 mg kg^−1^ (Fig. 7B), with no obvious change (4.67%), which was attributed to the lower adsorption capacity of Mg/Fe-LDH onto 2,4-D. It indicated that the loss control of 2,4-D by Mg/Al-LDH was higher than that of Mg/Fe-LDH in soil, which was also consistent with the results of adsorption kinetics studies.

## Conclusion

This study compared the adsorption capacity and intrinsic mechanism of Mg/Al-LDH and Mg/Fe-LDH to 2,4-D and provided novel absorbents for loss control of 2,4-D from soil. The adsorption of LDHs to 2,4-D was pH sensitive, and the adsorption capacity is negatively correlated with pH. Combined with various characterization technologies, the adsorption mechanisms of 2,4-D onto Mg/Al-LDH and Mg/Fe-LDH include electrostatic attraction and inner-sphere complexation mainly. However, the different 2,4-D adsorption capactiy between Mg/Al-LDH and Mg/Fe-LDH resulted from their differences in metal ion radius, surface charge density and adsorption energy (*E*_ads_). Mg/Al-LDH contains smaller metal ion radius, which provides greater surface charge density, resulting in stronger electrostatic attraction and inner spere complexation to 2,4-D than Mg/Fe-LDH. Although Mg/Fe-LDH could provide more specific surface area and have better structure for physical adsorption than Mg/Al-LDH, the adsorption capacity of Mg/Fe-LDH to 2,4-D was lower, indicating that chemical adsorption instead of physical adsorption was dominant. This conclusion was also supported by the results of the DFT calculation. The greater adsorption capacity of Mg/Al-LDH for 2,4-D was driven by the higher *E*_ads_ and lower electron density. Furthermore, the soil column leaching experiment proved that both LDHs had great loss control of 2,4-D in soil, and Mg/Al-LDH had a stronger effect than Mg/Fe-LDH. This study developed sustainable strategies to synthesize LDHs to reduce the loss of 2,4-D from soil and increase its bioavailability. Future research could be conducted in field experiments to investigate the effect of LDHs in actual natural conditions.

## Supplementary Information

Supplementary Material 1.

## Data Availability

All data generated or analysed during this study are included in this published article [and its supplementary information files].
