# Supplementary material for: Comparative study of Mg/Al-LDH and Mg/Fe-LDH on adsorption and loss control of 2,4-dichlorophenoxyacetic acid
Source: Adv Biotechnol (Singap). 2025 Jan 21;3(1):4. doi: 10.1007/s44307-024-00055-3 (PMC12443660; doi:10.1007/s44307-024-00055-3)
Supplement: Supplementary file 1 — Supplementary Material 1. [file 44307_2024_55_MOESM1_ESM.docx]

**Comparative study of Mg/Al-LDH and Mg/Fe-LDH on adsorption and loss control of 2,4-dichlorophenoxyacetic acid**

Zeyuan Zhang, Liangjie Tang, Jing Luo, Jinfang Tan, Xiaoqian Jiang*

School of Agriculture and Biotechnology, Sun Yat-sen University, Shenzhen, Guangdong 518107, PR China

*Corresponding to: [jiangxq7@mail.sysu.edu.cn](mailto:jiangxq7@mail.sysu.edu.cn.)

Contents

[Text 1 DFT calculation details 1](#_Toc179731975)

[Text 2 Zeta potentials 1](#_Toc179731975)

[Figure S1 XPS patterns of Mg/Al-LDH and Fe/Al-LDH before and after adsorption of 2,4-D at pH=5, 7, and 8 (MALD: Mg/Al-LDH after adsorption of 2,4-D; MFLD: Mg/Fe-LDH after adsorption of 2,4-D) 2](#_Toc179731973)

[Table S1 The proportion of elements in Mg/Al-LDH and Fe/Al-LDH before and after adsorption of 2,4-D at pH=5, 7, and 8 3](#_Toc179731974)

[Table S2 The fitting parameters of intra-particle diffusion model for adsorption experiments 3](#_Toc179731975)

[Table S3 Comparison of adsorption properties of conventional materials for 2,4-D in aqueous phase 3](#_Toc179731975)

**Text S1 DFT calculation details**

The study used Vienna Ab-initio Simulation Package (VASP) to perform density functional theory calculations and full geometry optimization of Mg/Al-LDH and Mg/Fe-LDH materials was carried out (Kresse and Furthmüller 1996; Blöchl 1994). The Perdew-Burke-Ernzerhof functional was employed for exchange-correlation effects (Perdew et al. 1996), while DFT+D3 was used for handling weak interactions (Grimme et al. 2010). The cut-off energy for the plane-wave basis was 450 eV. K-points were 2*2*1 in the Brillouin zone. 15 Å of layer vacuum was applied at Z-axis of slab models to avoid the Periodic effect. Energy and maximum stress were converged to 10^-5^ eV and 0.02 eV/Å, respectively. Adsorption energy was calculated by equation:

$E_{\mathrm{ads}}=E_{LDHs+2,4-D}-E_{\mathrm{LDHs}}-E_{2,4-D}$

where *E*_ads_ was the adsorption energy, *E*_LDHs+2,4-D_ was the total energy after the 2,4-D were adsorbed by Mg/Al-LDH or Mg/Fe-LDH. *E*_LDHs_ was the energy of Mg/Al-LDH or Mg/Fe-LDH and *E*_2,4-D_ was the energy of the 2,4-D.

Reference

Binh Q A, Nguyen H H. Investigation the isotherm and kinetics of adsorption mechanism of herbicide 2,4-dichlorophenoxyacetic acid (2,4-D) on corn cob biochar [J]. Bioresour. Technol. Rep., 2020, 11: 100520. https://doi.org/10.1016/j.biteb.2020.100520

Blöchl P E. Projector augmented-wave method [J]. Physical Review B - Condens Matter, 1994, 50(24): 17953-79. https://doi.org/10.1103/physrevb.50.17953

Calisto J S, Pacheco I S, Freitas L L, et al. Adsorption kinetic and thermodynamic studies of the 2, 4-dichlorophenoxyacetate (2,4-D) by the [Co–Al–Cl] layered double hydroxide [J]. Heliyon, 2019, 5(12): e02553. https://doi.org/10.1016/j.heliyon.2019.e02553

Grimme S, Antony J, Ehrlich S, et al. A consistent and accurate ab initio parametrization of density functional dispersion correction (DFT-D) for the 94 elements H-Pu [J]. The Journal of Chemical Physics, 2010, 132(15): 154104. https://doi.org/10.1063/1.3382344

Kresse G, Furthmüller J. Efficient iterative schemes for ab initio total-energy calculations using a plane-wave basis set [J]. Physical Review B - Condens Matter, 1996, 54(16): 11169-86. https://doi.org/10.1103/physrevb.50.17953

Legrouri A, Lakraimi M, Barroug A, et al. Removal of the herbicide 2,4-dichlorophenoxyacetate from water to zinc–aluminium–chloride layered double hydroxides [J]. Water Res., 2005, 39(15): 3441-8. https://doi.org/10.1016/j.watres.2005.03.036

Perdew J P, Burke K, Ernzerhof M. Generalized gradient approximation made simple [J]. Physical Review Letters, 1996, 77(18): 3865-8. https://doi.org/10.1103/PhysRevLett.77.3865

Salman J M, Njoku V O, Hameed B H. Batch and fixed-bed adsorption of 2,4-dichlorophenoxyacetic acid onto oil palm frond activated carbon [J]. Chem. Eng. J., 2011, 174: 33-40, https://doi.org/10.1016/j.cej.2011.08.024

**Text S2 Zeta potentials**

30 mL 2,4-D solution with concentrations of 0, 10, 25, and 50 mg L^-1^ in 0.01 mol L^-1^ NaCl background solution were prepared. The 0.003 g of LDH was added to the solution and the pH is adjusted to 3, 4, 5, 6, 7, 8, 9, 10, 11, 12, and 13, respectively. Finally, the sample was fully shaken for 24 h before the measurement was done.


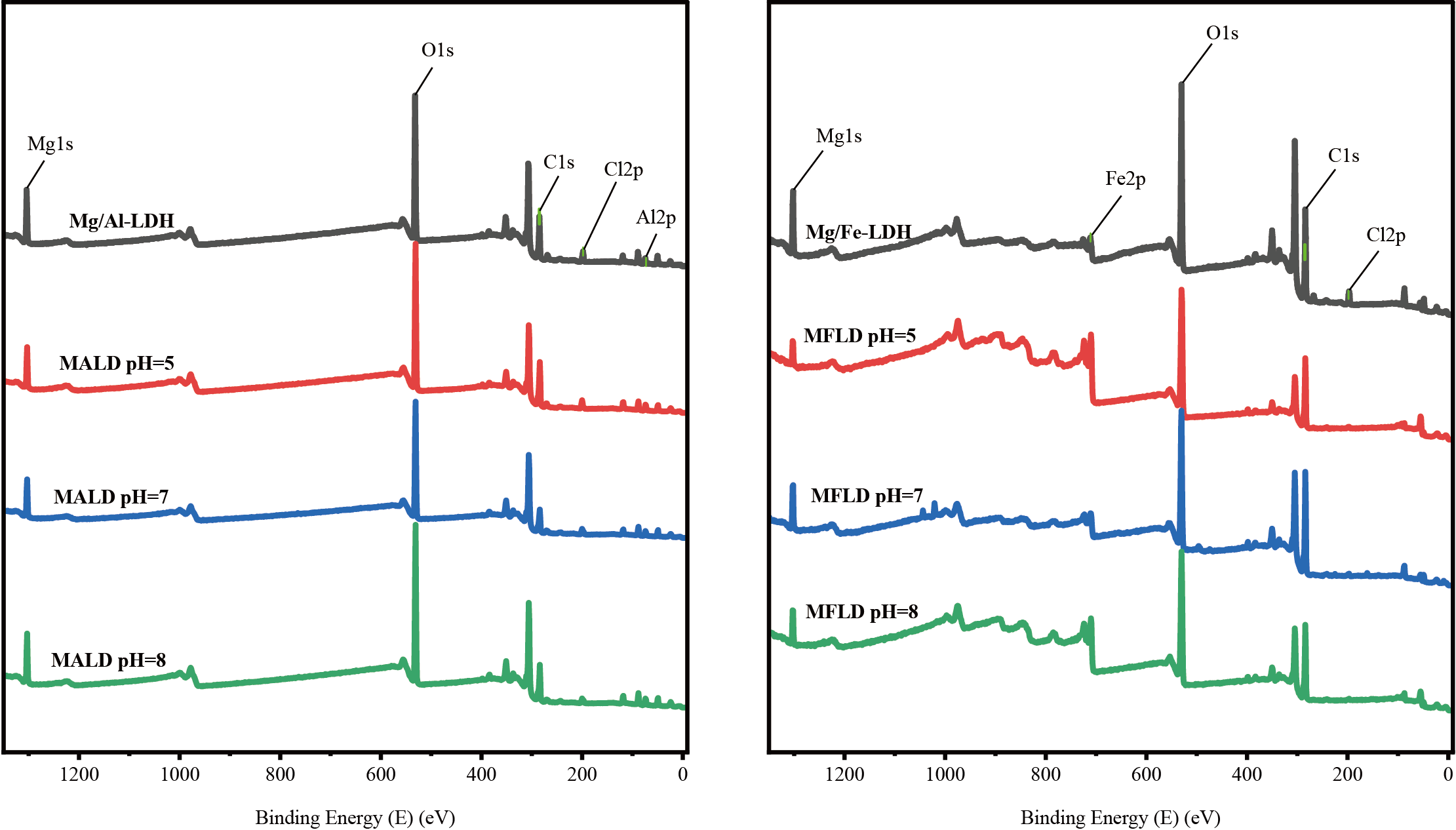


# Figure S1 XPS patterns of Mg/Al-LDH and Mg/Fe-LDH before and after adsorption of 2,4-D at pH=5, 7, and 8 (MALD: Mg/Al-LDH after adsorption of 2,4-D; MFLD: Mg/Fe-LDH after adsorption of 2,4-D)

# Table S1 The proportion of elements in Mg/Al-LDH and Mg/Fe-LDH before and after adsorption of 2,4-D at pH=5, 7, and 8

| Type of materials | Element content (%) | | | | | |
| --- | --- | --- | --- | --- | --- | --- |
|  | Mg(%) | Al(%) | Fe(%) | C(%) | O(%) | Cl(%) |
| Mg/Al-LDH | 7.98 | 6.81 | —— | 32.38 | 38.07 | 2.77 |
| MALD pH=5 | 6.75 | 8.02 | —— | 35.78 | 43.69 | 5.76 |
| MALD pH=7 | 8.67 | 8.43 | —— | 31.07 | 47.75 | 4.08 |
| MALD pH=8 | 8.12 | 9.54 | —— | 31.46 | 47.74 | 3.14 |
| Mg/Fe-LDH | 6.36 | —— | 13.01 | 44.91 | 33.39 | 2.33 |
| MFLD pH=5 | 3.59 | —— | 12.06 | 42.91 | 38.97 | 2.37 |
| MFLD pH=7 | 5.79 | —— | 4.35 | 53.2 | 33.99 | 2.67 |
| MFLD pH=8 | 4.91 | —— | 9.38 | 46.34 | 36.78 | 2.59 |

(MALD: Mg/Al-LDH after adsorption of 2,4-D; MFLD: Mg/Fe-LDH after adsorption of 2,4-D)

# Table S2 The fitting parameters of intra-particle diffusion model for adsorption experiments

| Adsorbent | First stage | | | Second stage | | | Third stage | | |
| --- | --- | --- | --- | --- | --- | --- | --- | --- | --- |
|  | *K_d1_* | *C_1_* | *R_1_^2^* | *K_d2_* | *C_2_* | *R_2_^2^* | *K_d3_* | *C_3_* | *R_3_^2^* |
| MALD pH=5 | 5.97 | 192.25 | 0.86 | 0.88 | 237.80 | 0.98 | 0.12 | 238.69 | 0.97 |
| MALD pH=7 | 5.64 | 178.96 | 0.95 | 1.50 | 208.12 | 0.91 | -0.81 | 233.98 | 0.32 |
| MALD pH=8 | 3.69 | 177.70 | 0.69 | -0.96 | 212.88 | - | -1.60 | 221.63 | - |
| MFLD pH=5 | 2.32 | 38.95 | 0.99 | 1.08 | 51.89 | 0.93 | 0.86 | 50.01 | 0.99 |
| MFLD pH=7 | 1.27 | 26.86 | 0.28 | -0.28 | 42.42 | 0.99 | 0.69 | 30.25 | 0.05 |
| MFLD pH=8 | 3.44 | -5.65 | - | 3.16 | -7.94 | 0.89 | 0.59 | 20.60 | 0.82 |

(MALD: Mg/Al-LDH after adsorption of 2,4-D; MFLD: Mg/Fe-LDH after adsorption of 2,4-D)

# Table S3. Comparison of adsorption properties of conventional materials for 2,4-D in aqueous phase.

| Material | C_0_(mg L^-1^) | pH | Adsorption capacity(mg g^-1^) | Reference |
| --- | --- | --- | --- | --- |
| [Co-Al-Cl] LDH | 60 | 5 | 3.8 | Calisto et al. 2019 |
| Zn-Al-Cl-LDH | 25 | 7 | 1.2 | Legrouri et al. 2005 |
| Corn cob biochar | 60 | 2 | 37.4 | Binh et al. 2020 |
| Oil palm frond biochar | 150 | 3.5 | 45.0 | Salman et al. 2011 |
| Mg/Al-LDH | 200 | 5 | 241.7 | This study |
| Mg/Fe-LDH | 200 | 5 | 64.0 | This study |
